# AI-Assisted Fatigue and Stamina Control for Performance Sports on IMU-Generated Multivariate Times Series Datasets

**DOI:** 10.3390/s24010132

**Published:** 2023-12-26

**Authors:** Attila Biró, Antonio Ignacio Cuesta-Vargas, László Szilágyi

**Affiliations:** 1 Department of Physiotherapy, University of Malaga, 29071 Malaga, Spain; acuesta@uma.es; 2 Department of Electrical Engineering and Information Technology, George Emil Palade University of Medicine, Pharmacy, Science, and Technology of Targu Mures, Str. Nicolae Iorga, Nr. 1, 540088 Targu Mures, Romania; 3 Biomedical Research Institute of Malaga (IBIMA), 29590 Malaga, Spain; 4 Faculty of Health Science, School of Clinical Science, Queensland University Technology, Brisbane 4000, Australia; 5 Physiological Controls Research Center, Óbuda University, 1034 Budapest, Hungary; szilagyi.laszlo@uni-obuda.hu; 6 Computational Intelligence Research Group, Sapientia Hungarian University of Transylvania, 540485 Targu Mures, Romania

**Keywords:** IMU, fatigue control, stamina, machine learning, deep learning, LSTM, assessment

## Abstract

Background: Optimal sports performance requires a balance between intensive training and adequate rest. IMUs provide objective, quantifiable data to analyze performance dynamics, despite the challenges in quantifying athlete training loads. The ability of AI to analyze complex datasets brings innovation to the monitoring and optimization of athlete training cycles. Traditional techniques rely on subjective assessments to prevent overtraining, which can lead to injury and underperformance. IMUs provide objective, quantitative data on athletes’ physical status during action. AI and machine learning can turn these data into useful insights, enabling data-driven athlete performance management. With IMU-generated multivariate time series data, this paper uses AI to construct a robust model for predicting fatigue and stamina. Materials and Methods: IMUs linked to 19 athletes recorded triaxial acceleration, angular velocity, and magnetic orientation throughout repeated sessions. Standardized training included steady-pace runs and fatigue-inducing techniques. The raw time series data were used to train a supervised ML model based on frequency and time-domain characteristics. The performances of Random Forest, Gradient Boosting Machines, and LSTM networks were compared. A feedback loop adjusted the model in real time based on prediction error and bias estimation. Results: The AI model demonstrated high predictive accuracy for fatigue, showing significant correlations between predicted fatigue levels and observed declines in performance. Stamina predictions enabled individualized training adjustments that were in sync with athletes’ physiological thresholds. Bias correction mechanisms proved effective in minimizing systematic prediction errors. Moreover, real-time adaptations of the model led to enhanced training periodization strategies, reducing the risk of overtraining and improving overall athletic performance. Conclusions: In sports performance analytics, the AI-assisted model using IMU multivariate time series data is effective. Training can be tailored and constantly altered because the model accurately predicts fatigue and stamina. AI models can effectively forecast the beginning of weariness before any physical symptoms appear. This allows for timely interventions to prevent overtraining and potential accidents. The model shows an exceptional ability to customize training programs according to the physiological reactions of each athlete and enhance the overall training effectiveness. In addition, the study demonstrated the model’s efficacy in real-time monitoring performance, improving the decision-making abilities of both coaches and athletes. The approach enables ongoing and thorough data analysis, supporting strategic planning for training and competition, resulting in optimized performance outcomes. These findings highlight the revolutionary capability of AI in sports science, offering a future where data-driven methods greatly enhance athlete training and performance management.

## 1. Introduction

**Figure 1:**
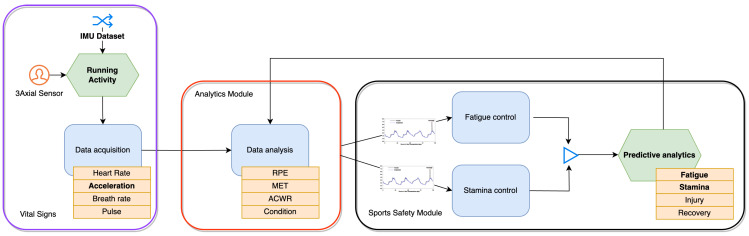
Sports safety approach on specific use case.

In the realm of sports and sports safety, the application of multivariate time series datasets is particularly pertinent due to the dynamic and multi-factorial nature of athletic performance and well-being [1]. These datasets consolidate multiple variables recorded sequentially over time, offering an integrative view that is instrumental in understanding and enhancing athletic performance while ensuring the safety of athletes (see Figure 1).

The interdependencies between various physiological and biomechanical factors are central to sports science [2]. For example, an athlete’s performance is not solely dependent on individual metrics like heart rate or speed but also on how these metrics interact. Multivariate time series analysis [3] allows sports scientists to simultaneously evaluate factors such as muscle fatigue, hydration levels, and biomechanical efficiency [4]. Such a comprehensive analysis is crucial, as it can inform tailored training regimens that optimize performance while mitigating the risk of injury. In sports safety, the synchronization of physiological events is crucial for injury prevention [5]. For instance, the concurrent analysis of joint stress, muscle activation, and impact forces can help in identifying patterns that precede common injuries. By understanding how these factors coalesce over time, interventions can be designed to reinforce athlete safety.

The complexity of an athlete’s adaptation to training over various time scales [6] also necessitates multivariate analysis [7,8]. Univariate time series might overlook patterns of overtraining or subtle signs of impending injury that only become evident when multiple variables are considered together. In sports medicine, monitoring an array of health indicators can provide a holistic view of an athlete’s recovery progress or readiness for competition, thereby facilitating personalized healthcare interventions [9]. Predictive modeling, powered by multivariate time series data, is invaluable in forecasting future performance and potential injury risks. By analyzing historical data across multiple dimensions of an athlete’s training load and physiological responses [10], predictive models can provide insights that guide training periodization, competition strategies, and injury prevention plans.

Moreover, nuanced anomaly detection within these datasets can preemptively alert coaches and medical staff to deviations from an athlete’s typical performance profile, which may indicate emerging health concerns or the onset of fatigue [11]. In high-performance sports, the ability to detect and respond to such anomalies promptly can be the difference between winning and sustaining an injury.

In terms of how they are used, multivariate time series datasets help improve complex AI models, like deep learning networks, which need a lot of different kinds of data to find hidden patterns [12]. These models are increasingly used to simulate complex athletic scenarios and to develop real-time decision-making tools for in-game strategy and training adjustments [13]. The advent of big data technologies has further empowered the sports industry to harness large-scale multivariate datasets for real-time analytics, providing a competitive edge and fortifying the safety protocols that protect athletes.

To summarize, multivariate time series datasets are very important in sports and sports safety [14] because they provide a solid foundation for creating advanced analytical tools that improve athletic performance and protect athlete health. Their comprehensive nature facilitates a deeper understanding of the multifaceted aspects of sports dynamics, making them an invaluable asset in the data-driven optimization of athletic training and care.

### 1.1. Fatigue and Stamina Calculation Algorithm

To include both fatigue and stamina calculations in a complex algorithm that uses deep learning (DL) and ensemble methods, a combined method would be needed that can tell the difference between the two states. First, we will represent the multivariate time series data from IMUs [15,16]. In algorithms, we can easily integrate a deep neural network (DNN) [17,18] that includes convolutional layers for spatial feature extraction and recurrent layers, like LSTM [19,20] or GRU, for temporal dependencies. The ensemble method for stamina and fatigue consists of various ML models such as gradient boosting, bagging, or additional neural networks (NNs) [21], each trained to predict stamina and fatigue from the high-level features learned by the deep learning model. The ensemble methods not only capture different aspects of the complex physiological data but also help to improve the robustness and accuracy of the predictions for stamina and fatigue. The feedback mechanism ensures that the models remain dynamic and adaptive, refining predictions as more data are collected over time.

### 1.2. Bias Calculation

Calculating bias in the context of stamina and fatigue detection [22] algorithms involves adjusting the predictive models to account for systematic errors that could affect their accuracy. This requires an understanding of how the models’ predictions deviate from the true values over a dataset. Firstly, X(t) will represent the multivariate time series data from IMUs, where *t* indexes time. The data include IMU sensor readings such as *acceleration* (*a*), *angular velocity* (ω), and *magnetic field orientation* (*m*) in three-dimensional space:

(1) $$X\left(t\right)=\left[\begin{array}{ccc} a_{x}\left(t\right) & a_{y}\left(t\right) & a_{z}\left(t\right)\\ \omega_{x}\left(t\right) & \omega_{y}\left(t\right) & \omega_{z}\left(t\right)\\ m_{x}\left(t\right) & m_{y}\left(t\right) & m_{z}\left(t\right) \end{array}\right]$$

Assuming we have predictive models for stamina and fatigue, we denote now the estimated values of stamina and fatigue at time *t* as $\hat{S}\left(t\right)$ and $\hat{F}\left(t\right)$, respectively. The true stamina and fatigue values are $S_{\mathrm{true}}\left(t\right)$ and $F_{\mathrm{true}}\left(t\right)$. The algorithm to calculate bias will be structured as follows: prediction generation for stamina and fatigue over a validation dataset *D* of size *N*:

(2) $$\hat{S}\left(t\right)=M_{\mathrm{stamina}}\left(X^{\prime }\left(t\right),\Theta_{\mathrm{stamina}}\right)$$

(3) $$\hat{F}\left(t\right)=M_{\mathrm{fatigue}}\left(X^{\prime }\left(t\right),\Theta_{\mathrm{fatigue}}\right)$$

where $M_{\mathrm{stamina}}$ and $M_{\mathrm{fatigue}}$ are the trained models, and $\Theta_{\mathrm{stamina}}$ and $\Theta_{\mathrm{fatigue}}$ are the model parameters. The bias for stamina ($B_{\mathrm{stamina}}$) is the average difference between the predicted and true stamina values:

(4) $$B_{\mathrm{stamina}}=\frac{1}{N}\sum_{t=1}^{N}\left(\hat{S}\left(t\right)-S_{\mathrm{true}}\left(t\right)\right)$$

Similarly, the bias for fatigue ($B_{\mathrm{fatigue}}$) is the average difference between the predicted and true fatigue values:

(5) $$B_{\mathrm{fatigue}}=\frac{1}{N}\sum_{t=1}^{N}\left(\hat{F}\left(t\right)-F_{\mathrm{true}}\left(t\right)\right)$$

If the bias is significant, the models’ predictions need to be updated to correct for the bias:

(6) $${\hat{S}}_{\mathrm{corrected}}\left(t\right)=\hat{S}\left(t\right)-B_{\mathrm{stamina}}$$

(7) $${\hat{F}}_{\mathrm{corrected}}\left(t\right)=\hat{F}\left(t\right)-B_{\mathrm{fatigue}}$$

After bias correction, the models need to be re-evaluated by using the corrected predictions to ensure the bias is minimized:

(8) $$\mathrm{Evaluate}({\hat{S}}_{\mathrm{corrected}}\left(t\right),S_{\mathrm{true}}\left(t\right))$$

(9) $$\mathrm{Evaluate}({\hat{F}}_{\mathrm{corrected}}\left(t\right),F_{\mathrm{true}}\left(t\right))$$

In this algorithm, the bias is quantified as the mean error across the dataset. If bias is detected, the models’ predictions are adjusted accordingly. This correction ensures that the models provide unbiased estimates of stamina and fatigue, which is critical for the accurate monitoring and adjustment of training regimens. Bias calculation and correction are essential steps in the deployment of predictive models to ensure that they perform well on new, unseen data and are robust across different populations and conditions.

## 2. Novelties of the Approach

**Figure 2:**
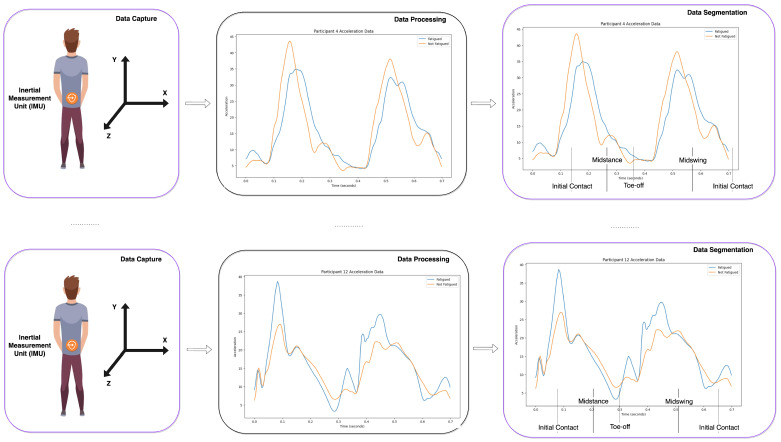
Data capturing and processing adapted from [27]. Data are captured by IMU and then segmented into individual strides.

The specificity of AI-assisted fatigue and stamina control in performance sports [23,24], utilizing IMU-generated multivariate time series datasets, lies in the precise and individualized insights it can provide, enabling a deeper understanding and more effective management of athlete performance. Several specific innovations are noteworthy:

1. *Granular biomechanical analysis* [25,26]: AI can dissect minute biomechanical changes that occur with fatigue, which are imperceptible to the human eye or traditional analysis (see Table 1). For instance, slight alterations in stride length, gait symmetry, or joint angles during a run could indicate the onset of fatigue (see Figure 2). By detecting these subtle biomechanical shifts, AI systems can signal the need for rest or technique adjustment to prevent injury.

2. *Fatigue profiling and benchmarking* [28]: ML models can be trained to establish a baseline profile of an athlete’s performance when they are non-fatigued. Subsequent performances can be continuously compared against this benchmark to detect deviations suggestive of fatigue. This approach allows for the customization of training loads and recovery times for each athlete.
3. *Stamina capacity modeling* [29]: Through longitudinal tracking, AI can model an athlete’s stamina capacity across different conditions and over time. This involves analyzing how long an athlete can maintain a certain intensity level before fatigue sets in, and how this capacity changes with different training regimens, environmental conditions, or stages of the competitive season.
4. *Predictive fatigue onset detection* [30]: by leveraging historical data, AI can predict when an athlete is likely to become fatigued in future training sessions or competitions. These predictions can inform the design of training sessions to avoid premature fatigue and can be used to optimize in-game strategies, such as player substitutions in team sports.
5. *Optimization of recovery protocols* [31,32]: AI can also monitor recovery, assessing whether an athlete’s biomechanical signals have returned to their baseline post-exertion. This information can guide the type and duration of recovery methods applied, such as active recovery, massage, or specialized nutrition intake.
6. *Enhanced load management* [33]: By continuously analyzing an athlete’s response to various training loads, AI can help in designing periodization programs that optimize performance while minimizing the risk of overtraining or under-training. Innovative research may yield predictive mechanisms for injury prevention, identifying the precursors to common training injuries and suggesting preventive measures.
7. *Customized tapering strategies* [34]: Pre-competition tapering is crucial for peak performance. AI systems can analyze how different tapering durations and intensities affect an athlete’s performance, enabling the creation of bespoke tapering strategies that are timed and scaled precisely for maximum effect (see Table 2).

These innovations represent a move toward a data-driven, personalized approach to athletic training and competition. By leveraging the specific, actionable insights provided by AI, sports professionals can make informed decisions that enhance performance, extend an athlete’s career, and transform the landscape of competitive athletics.

## 3. Objectives

The objectives of this research are to leverage AI and IMU-generated datasets to bring forth a paradigm shift (see Table 1) in how athlete fatigue and stamina are understood and managed, leading to more effective and personalized approaches in sports performance enhancement [35]. The research focused on AI-assisted fatigue and stamina control for performance sports [36] using IMU-generated multivariate time series datasets, which aim to improve athlete performance and welfare through innovative AI applications. These objectives can be broadly categorized into the development, validation, and implementation of a predictive model informed by high-dimensional and temporally rich datasets.

1. *Methodological contributions*: This research seeks to contribute methodologically to the fields of sports science, sports safety, and AI (see Table 2). It aims to demonstrate the application of cutting-edge AI techniques in interpreting high-dimensional physiological data, potentially setting new standards for research and practice in sports performance analytics.
2. *Development of predictive models*: The paper aims to develop advanced predictive models that integrate ML algorithms capable of processing and interpreting complex multivariate time series data. The goal is to identify and quantify the latent variables and intricate patterns that signal the onset of fatigue and changes in stamina, which are often imperceptible to human observation.
3. *Real-time monitoring and analysis*: One central objective is to enable real-time monitoring and analysis of athlete performance using IMUs [37], which continuously collect data across multiple axes. The research seeks to harness these data to provide immediate feedback on an athlete’s biomechanical and physiological state, facilitating timely interventions to manage fatigue and stamina during both training and competitive events.
4. *Personalization of training regimens*: The research endeavors to personalize training regimens by utilizing the predictive models to tailor training and recovery programs to individual athletes’ needs. By considering each athlete’s unique physiological responses, the aim is to optimize training loads and recovery periods, thus maximizing performance outcomes while minimizing the risk of injury and overtraining.
5. *Enhancement of recovery strategies*: Enhancing recovery strategies through precise predictions of fatigue levels is another key objective. By accurately forecasting recovery timelines, the model would inform the development of individualized recovery protocols, integrating modalities such as nutrition, physiotherapy, and rest.
6. *Strategic athletic planning*: Strategic planning for athletic training and competition schedules constitutes a further objective. The research intends to provide coaches and athletes with strategic tools to plan training cycles, tapering phases, and competition pacing strategies based on predictive insights into fatigue and stamina.

In the longer term, in collaboration with University teams (e.g., Toyo University from Japan) and by using the Sunbears Ecosystem [38] we plan to implement a program that delivers the improvement of athlete health and performance longevity. Improving overall athlete health and extending the longevity of their performance careers through a data-driven approach to training load management is a critical objective. By continuously adapting to the athlete’s physical state, the AI-assisted model aims to support sustainable high-performance training practices.

## 4. Materials and Methods

The development of fully adaptive real-time systems that can adjust training prescription instantaneously based on continuous data streams represents a significant future direction. These systems would operate as intelligent digital coaches, offering moment-to-moment guidance. Customized models that adapt to the individual characteristics of athletes, accounting for genetic factors, personal health history, and psychological state, could offer personalized training at an unprecedented level.

### 4.1. Data

This study utilized an openly accessible, validated multivariate time series dataset [27,39], derived from inertial measurement units (IMUs) to monitor kinematic patterns of 19 habitual, injury-free runners under conditions of fatigue and non-fatigue. The study protocol connected to the dataset used [39] was reviewed and approved by the human research ethics committee at University College Dublin, Ireland.

**Figure 3:**
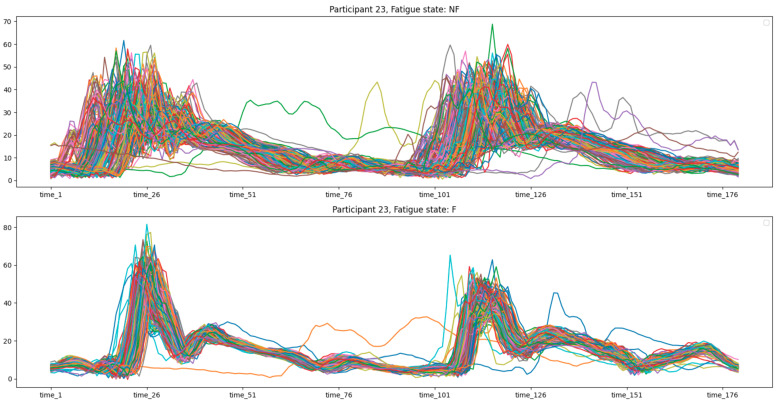
Participant 23—non-fatigue (NF) and fatigue (N) states. Details of more participants: See data availability statement.

Data acquisition was segmented into three distinct phases. Initially, subjects engaged in a 400-m run executed at a self-determined comfortable pace. Subsequent to this, a standardized fatigue-inducing protocol was administered, known as the beep test, which necessitates incremental running between two points spaced 20 m apart in synchrony with audio cues that decrease in intervals over time. This protocol escalates in intensity until the participant is unable to maintain the requisite pace. The concluding phase involved replicating the initial 400-m run, albeit under the influence of fatigue. Throughout these trials, the IMUs recorded triaxial measurements of acceleration, angular velocity, and magnetic orientation at a frequency of 256 samples per second. The protocol (also called the fatiguing protocol) concludes when the runner is no longer able to match the escalating speed. All the runs were conducted on an open-air running track [27]. The compiled dataset encompasses segmented strides from both 400-m runs across the entire cohort. Each data point is annotated with an identifier for the participant and a binary label indicating the presence (’F’) or absence (’NF’) of fatigue. One example of Participant’s visualization multivariate times series is visible in Figure 3. The dataset also incorporates three-dimensional accelerometer and gyroscope readings, with the orientation of each axis being relative to the sensor’s position. Additionally, composite signals representing overall acceleration and gyroscope magnitude were computed from the primary measurements and factored into the subsequent analysis [39].

### 4.2. Experimental Environment

The modeling and AI experiments were run on the following configurations: Mac Studio (Apple M1 Ultra, 64 GB Memory) and MacBook Pro (Apple M1 Max, 64 GB of memory) on the Google Colab Pro platform [5].

**Figure 4:**
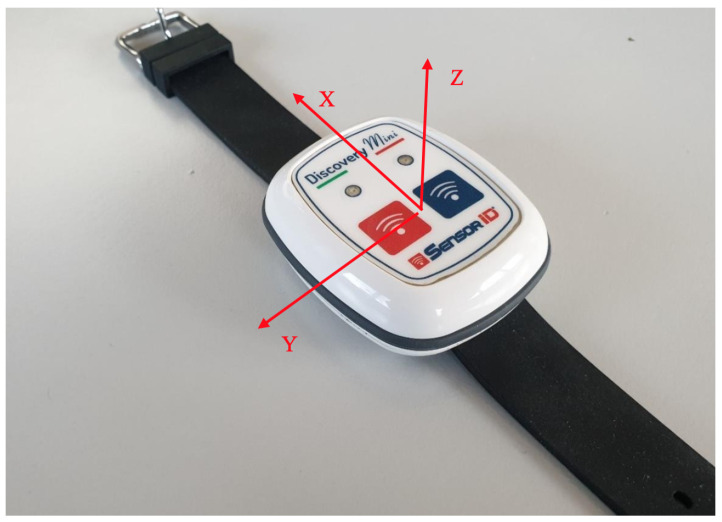
The Discovery Mini inertial sensor, showing the 3-axes.

For the captured dataset [39] Shimmer 3 IMU was used, installed on the lumbar of athletes [27]. For modeling and validation, this study first utilised the ActiGraph GT9X wireless inertial sensor [16], followed by an ultralow-power, high-performance, three-axis linear accelerometer [37] (Discovery Mini, using Lis2DH12) with inbuilt EEPROM memory (see Figure 4). The accelerometer had notification capabilities such as a buzzer or vibration characteristics. The device had a battery capacity of 400 milliampere-hours (mAh). The Discovery Mini IMU operates within a sampling frequency spectrum ranging from 1 Hz to 5.3 kHz. This sensor is proficient in measuring tri-axial acceleration (X, Y, Z axes) with a dynamic range extending up to ±16 g. For the purpose of this study, the sampling frequency was preset at 30 Hz to facilitate optimal data recording. Data extraction was conducted in its raw format, thereby preserving the integrity of the measured parameters. The data were extracted directly in raw format. We captured the sensor data in m/s${}^{2}$ and mg units. The sensor indicates the gravitational acceleration of Earth (9.8 m/s${}^{2}$). The data captured by the sensor are sent to the computer in real time by using Bluetooth Low Energy (BLE) protocol [37].

### 4.3. 3-Axial Accelerometer Sensors and IMUs in Performance Sports

IMU-generated multivariate time series datasets ensure AI-assisted fatigue and stamina regulation, a new performance sports paradigm with predictive precision and personalized training insights. The IMUs and 3-axial accelerometer sensors [16,37] can identify fatigue onset before symptoms appear, allowing for preemptive training modifications to improve performance and reduce injury risk. Multiple synchronized sensors provide a detailed profile of an athlete’s biomechanical functioning [40], including movement patterns and physiological reactions. This novel approach also promotes dynamic, real-time models that adapt to athlete conditioning and performance. Adding AI to this field also means a move toward coaching methods that are based on data, rather than qualitative observation. In these methods, decisions are made on the basis of continuous quantitative data. AI in multivariate time series dataset analysis will transform sports science by providing unprecedented personalization and precision in stamina and fatigue management optimization.

#### 4.3.1. Why Multivariate Times Series Datasets?

**Figure 5:**
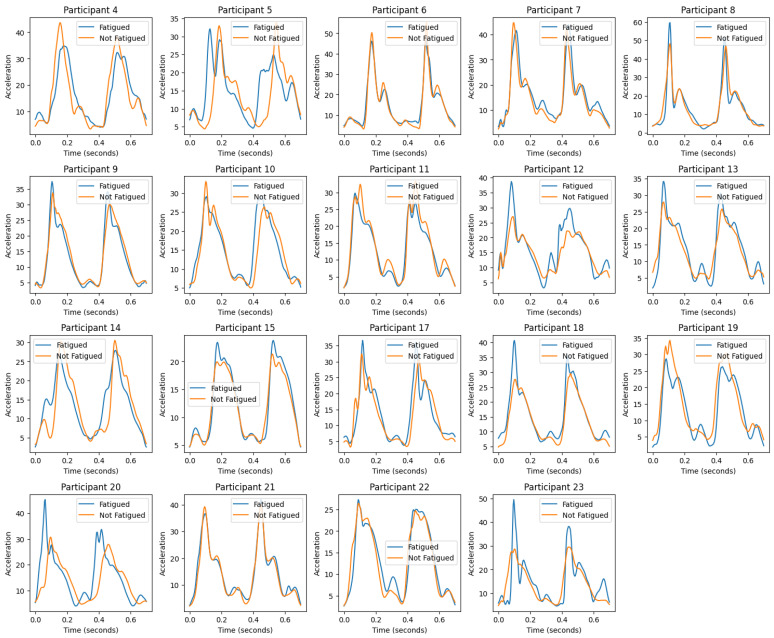
Acceleration data per specific segment for analysis.

Multivariate time series datasets represent a significant advancement in the field of AI, particularly due to their complex and rich nature. These datasets are composed of multiple variables or sequences recorded over time, which can provide a comprehensive view of the system being studied. Their utilization in AI projects brings several added values:

1. *Holistic approach*: Multivariate time series data enable the simultaneous analysis of multiple interconnected variables (see Figure 5). This approach facilitates a deeper understanding of complex systems, such as human physiology or financial markets, where the interaction between different factors is often as important as the individual variables themselves. In the context of recommendation systems, multivariate time series allow for the customization of user experiences by analyzing user interaction over time across various features, such as purchase history, browsing behavior, and search patterns.
2. *Improved prediction accuracy*: AI models trained on multivariate datasets can leverage the relationships between different variables to improve prediction accuracy. For instance, in weather forecasting, temperature, humidity, and wind speed work in concert to determine weather patterns, allowing for more accurate and reliable predictions.
3. *Advanced anomaly detection* [41]: By monitoring several indicators over time, AI systems can more effectively detect anomalies. In healthcare, for example, simultaneous analysis of multiple vital signs can help in the early detection of critical conditions, leading to timely interventions. The ability to forecast future trends based on historical multivariate data can significantly enhance risk management, particularly in the finance and insurance industry, by predicting the likelihood of adverse events and their potential impacts.
4. *Dynamic system insights and dynamic decision-making*: Multivariate datasets are instrumental in understanding dynamic systems. AI models can capture temporal dependencies and changes over time, offering insights into the evolution of the system’s state and facilitating dynamic decision-making. Analyzing data across multiple time frames allows AI to understand short-term fluctuations versus long-term trends, which is crucial for strategic planning in business and policy-making.
5. *Complex feature interactions* [42,43]: AI models can discover non-linear interactions and dependencies between features that may not be apparent in univariate analysis. This capability is crucial in fields like genomics, where the interaction between genes can influence the traits and health of an organism.
6. *Robustness to missing or partial data*: With multiple variables, AI techniques can better handle missing data by imputing missing values based on observed correlations in the dataset. This robustness is especially important in real-world scenarios where data collection may be imperfect.

In conclusion, multivariate time series datasets are invaluable in enhancing the depth, accuracy, and operational efficiency of AI models across various disciplines, providing a multi-dimensional perspective that captures the complexity of real-world phenomena.

#### 4.3.2. Fatigue Detection on IMU-Generated Multivariate Times Series Dataset

The specific algorithm to detect fatigue from IMU-generated multivariate time series datasets involves the following steps: We introduce X(t), which represents the multivariate time series data obtained from the IMUs, where *t* indicates the time step. The data could include, for instance, acceleration, angular velocity, and magnetometer data along three axes, such that:

(10) $$X\left(t\right)=\left[\begin{array}{ccc} a_{x}\left(t\right) & a_{y}\left(t\right) & a_{z}\left(t\right)\\ \omega_{x}\left(t\right) & \omega_{y}\left(t\right) & \omega_{z}\left(t\right)\\ m_{x}\left(t\right) & m_{y}\left(t\right) & m_{z}\left(t\right) \end{array}\right]$$

where *a* represents acceleration, ω represents angular velocity, and *m* represents magnetic field orientation, each along the *x*, *y*, and *z* axes respectively. It will be followed by the normalization and IMU filter to remove noise and artifacts:

(11) $$X^{\prime }\left(t\right)=f\left(X\left(t\right)\right)$$

where *f* represents a series of preprocessing functions such as filtering and normalization. This is followed by feature extraction, where the relevant features F are data indicative of fatigue from the preprocessed data:

(12) $$F\left(t\right)=g(X^{\prime }\left(t\right))$$

where *g* is a function that could include statistical measures, frequency domain analysis, or other feature extraction methods. After this, we define a predictive model *M*, which relates the features F to a binary fatigue state Y(t), where Y(t)∈{0,1} indicates non-fatigued and fatigued states, respectively:

(13) Y(t)=M(F(t),Θ)

where Θ represents the parameters of the model that are learned during training. On model training, we utilize a labeled dataset $D={\left\{\left(F_{i},Y_{i}\right)\right\}}_{i=1}^{N}$, where *N* is the number of samples, to learn the model parameters Θ using a suitable learning algorithm *L*:

(14) $$\Theta^{*}=L\left(D\right)$$

The algorithm will then apply the trained model to new IMU data to detect fatigue:

(15) $$\hat{Y}\left(t\right)=M\left(F\left(t\right),\Theta^{*}\right)$$

where $\hat{Y}\left(t\right)$ is the predicted fatigue state. Finally, a thresholding function *h* was implemented to determine fatigue:

(16) $$\mathrm{Fatigue}\left(t\right)=\left\{\begin{array}{cc} 1 & \mathrm{if}\;h(\hat{Y}\left(t\right))\geq \tau \\ 0 & \mathrm{otherwise} \end{array}\right.$$

where τ is a predefined threshold that discriminates between fatigued and non-fatigued states. The specific form of the functions *f*, *g*, *M*, and *L*, as well as the threshold τ, were determined by the characteristics of the dataset, the nature of the sporting activity, and the performance objectives of the algorithm. AI models such as DNN encapsulate most of these steps within their architecture, automating feature extraction and prediction through end-to-end learning.

#### 4.3.3. Stamina Detection on IMU-Generated Multivariate Times Series Dataset

To delineate a more specific algorithm for detecting stamina by using IMU-generated multivariate time series datasets [44,45], the process needs to be refined with additional mathematical constructs and data science terminologies that cater to this context. Stamina here is quantified as a measure of an athlete’s capacity to sustain optimal performance levels over time. Given a multivariate time series dataset X(t) from an IMU, we first defined the dataset more formally:

(17) $$X\left(t\right)=\left\{x_{1}\left(t\right),x_{2}\left(t\right),\ldots ,x_{p}\left(t\right)\right\}$$

where each $x_{i}\left(t\right)$ is a vector containing the *i*th sensor’s readings at time *t*, and *p* is the total number of sensors. The data preprocessing (P) phase transforms the raw IMU data to reduce noise and correct for bias and drift:

(18) $$X^{\prime }\left(t\right)=P\left(X\left(t\right)\right)$$

This may include techniques, such as Butterworth filtering, detrending, and normalization procedures to standardize sensor readings [46,47]. On feature extraction (F), from the preprocessed data, extracting features thought to correlate with stamina potentially involves: (1) time-domain features such as mean, median, variance, and root mean square of sensor signals; (2) frequency-domain features obtained via fast Fourier transform (FFT) or power spectral density (PSD) analysis; as well as the (3) entropy-based features that may indicate physiological complexity and resilience.

(19) $$F\left(t\right)=F(X^{\prime }\left(t\right))$$

We employ a deep learning architecture (D) to model complex relationships and temporal dependencies:

(20) H(t)=D(F(t),Φ)

where H(t) represents high-level features learned by the deep learning model (e.g., a Long Short-Term Memory (LSTM) network [48]), and Φ denotes the network parameters. We integrate an ensemble of models (E) that captures different aspects of the data and learns diverse representations:

(21) Y(t)=E(H(t),Ψ)

where Y(t) is the ensemble prediction for stamina, and Ψ represents the ensemble parameters. The ensemble may include models like Random Forests, Gradient Boosting Machines, or additional neural networks. The model training and optimization (O) phase will adjust the parameters of the deep learning model and the ensemble method by minimizing a loss function over a labeled dataset:

(22) $$\Phi^{*},\Psi^{*}=\underset{\Phi ,\Psi }{\mathrm{argmin}}\;L\left(Y\left(t\right),Z_{\mathrm{true}}\left(t\right)\right)$$

where $Z_{\mathrm{true}}\left(t\right)$ represents the ground truth stamina levels, and L is a suitable loss function, such as cross-entropy for classification tasks or mean squared error for regression tasks. Now we can apply dynamic time warping (DTW) [49], which helps us to compare time series of varying lengths (as stamina exercises might vary in duration). The DTW for alignment is defined as:

(23) $$F_{\mathrm{aligned}}\left(t\right)=\mathrm{DTW}\left(F\left(t\right)\right)$$

The stamina metric definition (*Z*) defines a continuous stamina metric based on physiological and performance thresholds, normalized to a suitable scale:

(24) $$Z\left(t\right)=Z(F_{\mathrm{aligned}}\left(t\right))$$

This function could be a linear combination or a more complex non-linear model calibrated through physiological studies. The model training and parameter estimation (M, Θ) will train a predictive model M by using a suitable algorithm (e.g., regression, support vector machine, neural networks) to relate the features F to the stamina metric *Z*:

(25) $$\Theta^{*}={\mathrm{argmin}}_{\Theta }L\left(M\left(F\left(t\right),\Theta \right),Z\left(t\right)\right)$$

where L is a loss function that quantifies the error in stamina estimation, and $\Theta^{*}$ represents the optimized model parameters. The stamina estimation ($\hat{Z}$) estimates the specific metric for new data by using the trained model:

(26) $$\hat{Z}\left(t\right)=M\left(F\left(t\right),\Theta^{*}\right)$$

After this, we categorize the stamina levels (T) by using predefined physiological thresholds based on normalized stamina metrics:

$$\mathrm{Stamina}\_\mathrm{Category}\left(t\right)=T(\hat{Z}\left(t\right))$$

where, T applies threshold values that segment the stamina metric into distinct categories, such as ’low’, ’medium’, and ’high’ endurance. Lastly, the validation of the model (V) predictions is executed with the actual athlete performance data, using metrics such as correlation coefficients or Bland–Altman plots to assess agreement:

(27) $$V(\hat{Z}\left(t\right),Z_{\mathrm{true}}\left(t\right))$$

where $Z_{\mathrm{true}}\left(t\right)$ are true stamina values obtained from performance tests or expert assessments. If Stamina_Category is not used, then the model validation (V) for predictive power using appropriate validation techniques such as k-fold cross-validation or time series cross-validation will be as follows:

(28) $$V(Y\left(t\right),Z_{\mathrm{true}}\left(t\right))$$

Finally, the feedback loop for real-time adaptation (FB) will adopt a feedback loop that allows the model to adapt based on real-time data, enhancing the model’s predictive accuracy over time:

(29) $$\Phi^{{*}^{\prime }},\Psi^{{*}^{\prime }}=\mathrm{FB}\left(\Phi^{*},\Psi^{*},\mathrm{new}\;\mathrm{data}\right)$$

In this algorithm, D could utilize architectures such as convolutional neural networks (CNNs) for spatial feature extraction from the IMU data [50,51,52], followed by LSTMs or gated recurrent units (GRUs) to capture temporal patterns. The ensemble E would harness the predictive capabilities of multiple models to improve generalizability and reduce the likelihood of overfitting to the training data. The combination of these advanced methodologies enables the detection of subtle, non-linear patterns within the physiological data, which are indicative of stamina, and provides a robust prediction mechanism adaptable to the evolving fitness levels of athletes. By incorporating domain-specific feature extraction and leveraging dynamic time warping for alignment, this algorithm will robustly estimate an athlete’s stamina. The use of a predictive model trained on labeled data allows for the quantification of stamina in a continuous manner, offering a detailed view of the athlete’s endurance capabilities over time. The final validation step ensures that the model’s estimates are reliable and reflective of true stamina levels.

#### 4.3.4. Stamina and Fatigue Control Model

Creating an algorithm to control stamina and fatigue during training sessions with IMU-generated multivariate time series datasets involves a complex interplay of signal processing, feature extraction, statistical modeling and sports management. The algorithm is expected to provide real-time feedback and adapt training intensity based on the athlete’s current stamina and fatigue levels. We use the X(t) matrix of sensor readings from the IMU at time *t*, encompassing acceleration (*a*), angular velocity (ω), and other measures like magnetic orientation (*m*) across three axes x,y,z, as defined previously. In data preprocessing (P) phase we apply the filtering and normalization to the raw IMU data:

(30) $$X^{\prime }\left(t\right)=P\left(X\left(t\right)\right)$$

This may include detrending, smoothing, or other techniques to correct for sensor bias and noise. This is followed by the feature extraction (F), which derives a feature set F(t) that captures aspects related to fatigue and stamina:

(31) $$F\left(t\right)=F(X^{\prime }\left(t\right))$$

The features include metrics such as the mean and variance of acceleration, signal magnitude area (SMA), and entropy measures. The fatigue and stamina indicators ($I_{\mathrm{fatigue}},I_{\mathrm{stamina}}$) will develop indicators for fatigue and stamina based on the following features:

(32) $$I_{\mathrm{fatigue}}\left(t\right)=I_{\mathrm{fatigue}}\left(F\left(t\right)\right)$$

(33) $$I_{\mathrm{stamina}}\left(t\right)=I_{\mathrm{stamina}}\left(F\left(t\right)\right)$$

These indicators reflect the current state of the athlete and are sensitive to changes over time. The training session control (C) formulates the control mechanisms that adjust training intensity based on fatigue and stamina levels:

(34) $$T\left(t\right)=C(I_{\mathrm{fatigue}}\left(t\right),I_{\mathrm{stamina}}\left(t\right))$$

where T(t) represents the training parameters to be adjusted, such as intensity, duration, or rest periods. The model training and parameter estimation (M, Θ) use the historical training data to train a model M, which correlates fatigue and stamina indicators with optimal training outcomes:

(35) $$\Theta^{*}={\mathrm{argmin}}_{\Theta }L\left(M\left(F\left(t\right),\Theta \right),O\left(t\right)\right)$$

where L is the loss function, O(t) represents the desired training outcomes, and $\Theta^{*}$ represents the optimized parameters. The feedback loop ($F_{\mathrm{loop}}$) implements a feedback loop to provide real-time adjustments:

(36) $$T^{\prime }\left(t+1\right)=F_{\mathrm{loop}}\left(T\left(t\right),\Delta I_{\mathrm{fatigue}}\left(t\right),\Delta I_{\mathrm{stamina}}\left(t\right)\right)$$

where Δ denotes the change in fatigue and stamina indicators, providing the basis for adjusting T for the next time step. Finally, the validation and adaptation (V, A) will continuously validate and adapt the control model:

(37) $$V(T^{\prime }\left(t\right),O\left(t\right))$$

(38) $$\Theta^{{*}^{\prime }}=A\left(\Theta^{*},V\right)$$

where V assesses the effectiveness of the training adjustments, and A modifies the model parameters Θ as needed for improved control. A complex ML model could be added into this algorithm M, which requires iterative updates as more data are collected. The control mechanism C is planned to be designed to ensure that training intensity does not exceed the athlete’s recovery capacity, thereby optimizing performance gains while minimizing the risk of overtraining. The feedback loop is crucial for adapting training parameters in real time based on the athlete’s responses, ensuring that each session is aligned with the athlete’s current physiological state.

#### 4.3.5. Bias Localization and Minimization

Incorporating a control function into the algorithm to localize and minimize biases in the prediction of stamina and fatigue from IMU-generated data requires an iterative process. This process involves identifying the source of the bias within the predictive models and iteratively refining the model to reduce this bias. First, X(t) denotes the multivariate time series data from IMUs, where $\hat{S}\left(t\right)$ and $\hat{F}\left(t\right)$ represent the models’ predictions for stamina and fatigue at time *t*, respectively, with $S_{\mathrm{true}}\left(t\right)$ and $F_{\mathrm{true}}\left(t\right)$ being the true values. The control function for bias localization and minimization can be outlined as follows. We can estimate the bias for stamina and fatigue predictions as:

(39) $$B_{\mathrm{stamina}}=\frac{1}{N}\sum_{t=1}^{N}\left(\hat{S}\left(t\right)-S_{\mathrm{true}}\left(t\right)\right)$$

(40) $$B_{\mathrm{fatigue}}=\frac{1}{N}\sum_{t=1}^{N}\left(\hat{F}\left(t\right)-F_{\mathrm{true}}\left(t\right)\right)$$

This will be followed by identifying patterns or features within X(t), which are consistently associated with bias in predictions, using a diagnostic analysis D:

(41) $$L_{\mathrm{stamina}}=D\left(X\left(t\right),B_{\mathrm{stamina}}\right)$$

(42) $$L_{\mathrm{fatigue}}=D\left(X\left(t\right),B_{\mathrm{fatigue}}\right)$$

$L_{\mathrm{stamina}}$ and $L_{\mathrm{fatigue}}$ represent localized features contributing to bias. The control function (C) to adjust model parameters or features to minimize bias is defined as:

(43) $$\Theta_{\mathrm{stamina}}^{\prime }=C\left(\Theta_{\mathrm{stamina}},L_{\mathrm{stamina}}\right)$$

(44) $$\Theta_{\mathrm{fatigue}}^{\prime }=C\left(\Theta_{\mathrm{fatigue}},L_{\mathrm{fatigue}}\right)$$

where $\Theta_{\mathrm{stamina}}^{\prime }$ and $\Theta_{\mathrm{fatigue}}^{\prime }$ are the adjusted model parameters. The main scope of the model update and the bias minimization mechanism is to update the models with the new parameters:

(45) $${\hat{S}}^{\prime }\left(t\right)=M_{\mathrm{stamina}}\left(X^{\prime }\left(t\right),\Theta_{\mathrm{stamina}}^{\prime }\right)$$

(46) $${\hat{F}}^{\prime }\left(t\right)=M_{\mathrm{fatigue}}\left(X^{\prime }\left(t\right),\Theta_{\mathrm{fatigue}}^{\prime }\right)$$

After this step, the bias will be recalculated to assess the effectiveness of the control function:

(47) $$B_{\mathrm{stamina}}^{\prime }=\frac{1}{N}\sum_{t=1}^{N}\left({\hat{S}}^{\prime }\left(t\right)-S_{\mathrm{true}}\left(t\right)\right)$$

(48) $$B_{\mathrm{fatigue}}^{\prime }=\frac{1}{N}\sum_{t=1}^{N}\left({\hat{F}}^{\prime }\left(t\right)-F_{\mathrm{true}}\left(t\right)\right)$$

Furthermore, an iterative refinement will be applied to the control function until the biases $B_{\mathrm{stamina}}^{\prime }$ and $B_{\mathrm{fatigue}}^{\prime }$ are minimized below the predefined threshold ϵ:

(49) $$\mathrm{while}\;(|B_{\mathrm{stamina}}^{\prime }\left|>\epsilon )\;\mathrm{or}\;(\right|B_{\mathrm{fatigue}}^{\prime }\left|>\epsilon \right):$$

(50) $$\;\Theta_{\mathrm{stamina}}^{\prime },\Theta_{\mathrm{fatigue}}^{\prime }=C\left(\ldots \right)$$

(51) Recalculatebiases(seeFormulas(47)and(48))

Once the biases are minimized, finalize the updated models for stamina and fatigue predictions. This algorithm incorporates an advanced control theory approach to systematically reduce bias in predictive modeling. The control function C is designed to be adaptive, capable of responding to the localized sources of bias and iteratively tuning the model parameters to address these issues. This process may involve techniques such as gradient descent for parameter adjustment or feature transformation methods to alter the input data space, thereby ensuring that the model’s outputs align closely with the true values.

#### 4.3.6. Training Session Duration and Intensity Optimization

As a long-term scope, to optimize training session duration and intensity while controlling for fatigue and stamina, and continuously keeping biases minimized, we need to integrate a control function into our algorithm that adjusts training parameters in response to real-time feedback from the athlete’s performance data. The aim is to achieve an optimal balance between exertion and recovery, ensuring that training is effective without leading to overtraining or under-training. The following presents an advanced algorithmic structure with an integrated control function for this purpose: X(t) represents the multivariate time series data from IMUs, with P(t) denoting the training parameters at time *t*, including duration d(t) and intensity i(t). The athlete’s real-time stamina and fatigue levels are estimated as $\hat{S}\left(t\right)$ and $\hat{F}\left(t\right)$, respectively, with biases $B_{\mathrm{stamina}}$ and $B_{\mathrm{fatigue}}$ calculated as previously outlined. The control function for optimizing training parameters can be structured as follows. First, we will establish initial training parameters based on the athlete’s baseline performance data.

(52) P(0)={d(0),i(0)}

On the planned training session execution, we will conduct the training session with the given parameters and collect IMU data.

(53) ExecuteTraining(P(t))

(54) X(t)=CollectData()

For a real-time prediction and bias correction functionality, stamina and fatigue were estimated, while applying the bias corrections from previous iterations at the same time.

(55) $${\hat{S}}_{\mathrm{corrected}}\left(t\right),{\hat{F}}_{\mathrm{corrected}}\left(t\right)=\mathrm{PredictAndCorrect}\left(X\left(t\right),B\right)$$

The control function for optimization ($C_{\mathrm{opt}}$) is defined based on the corrected stamina and fatigue estimates, adjusting the training parameters:

(56) $$P^{\prime }\left(t+1\right)=C_{\mathrm{opt}}\left({\hat{S}}_{\mathrm{corrected}}\left(t\right),{\hat{F}}_{\mathrm{corrected}}\left(t\right),P\left(t\right)\right)$$

The step is followed by a recalculation of biases based on the outcomes of the optimized training session:

(57) $$B^{\prime }=\mathrm{RecalculateBiases}\left(X\left(t\right),P^{\prime }\left(t+1\right)\right)$$

As next step, it is called the adaptive parameter update to facilitate the training parameters for the next session using the control function outputs and recalculated biases:

(58) $$P\left(t+1\right)=\mathrm{UpdateParameters}(P^{\prime }\left(t+1\right),B^{\prime })$$

**Figure 6:**
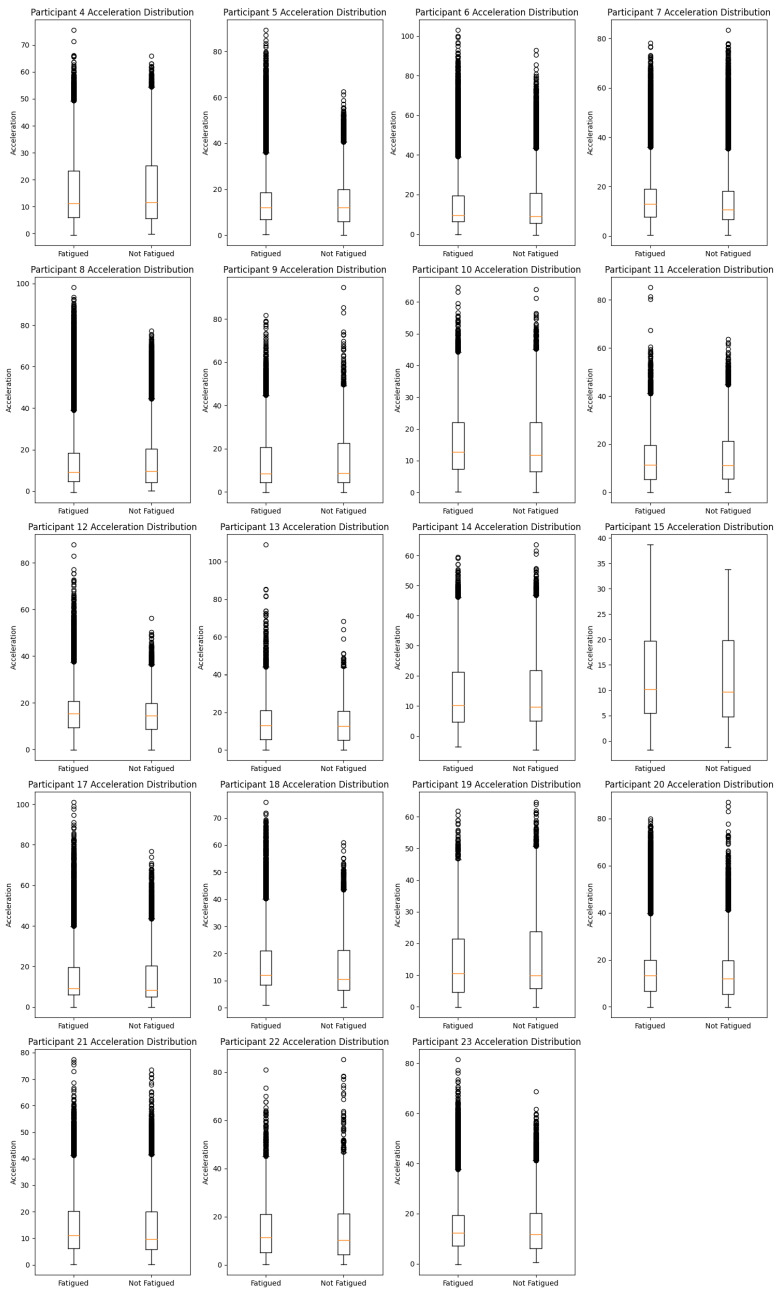
Acceleration distribution per participant.

The iterative loop is applied to perform steps 2–6 for subsequent training sessions, continuously adjusting for biases and optimizing training parameters. The control function $C_{\mathrm{opt}}$ could use optimization techniques such as (1) Model Predictive Control (MPC): A form of control that uses a model of the athlete’s response to training to predict future states and make adjustments to optimize the training (see Figure 6) load; (2) Reinforcement Learning (RL): An area of ML concerned with how software agents ought to take actions in an environment to maximize the notion of cumulative reward, which could be the athlete’s performance metrics. The algorithm should also incorporate a learning rate α that determines the magnitude of adjustments to the training parameters to avoid drastic changes that could harm the athlete. The continuous recalibration of biases ensures that the model remains accurate over time, even as the athlete’s fitness level changes. This adaptive, data-driven approach aims to personalize training regimens for optimal performance outcomes.

### 4.4. Comparison against Gold-Standard Methods

Within the domain of performance sports, the most reliable techniques for evaluating athlete tiredness and endurance have typically relied on physiological evaluations (see Table 3), including VO2 max tests, lactate threshold measures, EMG data, and subjective scales, such as the Rate of Perceived Exertion (RPE). Although these methods have been verified and proven to be trustworthy, they generally involve intrusive procedures, they require a significant amount of time, and the correlation with kinematic data is strong [53]. Kinematic measurements are widely used by physical therapists and sports trainers to quantify fatigue and injuries [54] and video-based optoelectronic systems are the gold standard; however, outside the lab, the IMU system is shown to be a solid alternative [55].

The emergence of AI-assisted methodologies, specifically those utilizing IMU-generated multivariate time series data, offers a novel alternative to these conventional approaches. The AI models enable the collection of data in a non-invasive and real-time manner, allowing for continuous monitoring of physiological and biomechanical indicators without significantly disturbing the athlete’s natural performance environment. AI-based models provide a continuous and dynamic examination of an athlete’s state, which allows for more accurate and timely detection of tiredness and changes in stamina compared to periodic assessments using gold-standard [56,57] approaches.

Although AI-assisted techniques show promising outcomes, such as the refined discernment of Quadratic Discriminant Analysis (QDA) or the high rates of correctly identifying relevant instances in Support Vector Machines (SVMs), they now serve as supplementary tools rather than complete substitutes for conventional measurements. The dataset exhibits accuracy values that consistently hover around the 50% threshold, suggesting that these approaches are still in their early developmental stages. Furthermore, it is crucial to thoroughly compare and evaluate the accuracy and effectiveness of AI techniques with established and highly reliable methodologies in order to determine their efficacy. Comparing traditional video-based analysis as the gold standard against IMUs integrated with AI-assisted methods (see Table 4) will reflect the specific contrasts between video-based techniques, traditionally considered the gold standard, especially in technique analysis and movement studies, and the emerging AI-driven approaches using IMUs for in-depth and continuous assessment of athletic performance. In conclusion, the comparison against gold standard methods demonstrates that AI-assisted techniques have the capacity to transform athlete monitoring by offering a consistent and comprehensive view of performance metrics. However, in order to match the effectiveness of established methods, it is crucial to further improve AI models to increase their ability to accurately anticipate outcomes. This will ensure that these new approaches can be relied upon as independent instruments in the high-pressure field of competitive sports.

## 5. Preprocessing

During the preprocessing phase, we localized and selected the athletes from the dataset. After that, we prepared a validation model, using the 3-axial accelerometers to model and/or cross-validate the experiments. Based on a standard beep test, the box plots show how the acceleration distribution changed during a 400-m run for 19 healthy, experienced runners when they were tired (F) and when they were not tired (NF). The acceleration data from each participant’s IMUs is split into “fatigued” and “not fatigued” states so that we can see how fatigue changes their kinematic patterns. Across box plots, there is a visible variation in the distribution of acceleration values between fatigued and not fatigued states. Most participants exhibit a higher median acceleration in the ’Not Fatigued’ state, suggesting a decrease in acceleration when fatigued. Additionally, wider boxes and longer whiskers indicate that some participants’ acceleration values spread out more when they are tired. This means that their performance is more variable when they are tired. The presence of outliers, particularly in the ’Fatigued’ condition, suggests that there are instances where the acceleration deviates significantly from the typical range. This could indicate sporadic bursts or drops in energy levels due to fatigue.

*Participant 4* shows a slight decrease in median acceleration when fatigued. The interquartile range (IQR) expands during fatigue, indicating increased variability in acceleration; *Participant 5* exhibits a notable reduction in median acceleration from ’Not Fatigued’ to ’Fatigued’. The presence of outliers in the ’Fatigued’ state suggests occasional deviations from typical performance levels; *Participant 7* has one of the biggest drops in median acceleration due to fatigue, along with a rise in IQR, which shows that fatigue has a big effect; *Participant 11* reveals a narrower IQR in the ’Fatigued’ state, which is unusual compared to other participants and may indicate a more consistent performance under fatigue; *Participant 13* has a median acceleration that remains relatively stable across states, suggesting resilience to the effects of fatigue; in case of *Participant 17*, like Participant 7, there is a big drop in the median acceleration when they are tired, along with a wide range of values showing that they are not performing consistently; *Participant 23* shows a significant number of outliers in the ’Fatigued’ state, suggesting erratic performance, which could signal problematic fatigue management.

Highlighting the *problematic participants* and *best performers*: *Problematic*: those with a substantial drop in median acceleration and increased variability during fatigue—such as Participants 7 and 17—are considered problematic as they exhibit a pronounced negative impact of fatigue on their performance; *Best results*: participants like 13, who maintain a stable median acceleration and a consistent performance range despite fatigue, can be categorized as the best performers in managing fatigue.

## 6. Experiments

The results (see Table 5) highlight the diverse capabilities and limitations of ML algorithms in predicting athletic fatigue and stamina. The moderate accuracy hovering around the 50% mark for most classifiers, including the *Extra Trees* and *Random Forest*, suggests that while these models can capture some patterns in the data, there is substantial room for improvement. The trade-off between precision and recall in these models is evident, with none excelling distinctly in both metrics, indicating challenges in achieving both high specificity and sensitivity. The *Quadratic Discriminant Analysis* (QDA) classifier shows a propensity for higher recall rates, suggesting it is particularly sensitive to true positive cases; however, its lower precision rate points to a higher rate of false positives. This could imply that QDA is better suited for scenarios where missing a true positive is more critical than falsely identifying a negative case as positive, which could be more applicable in early screening or diagnostic settings rather than precise performance prediction. In contrast, the *K-Nearest Neighbor* (KNN) and *Decision Tree* classifiers, with their lower recall rates, indicate a tendency to miss true positives more often, which could be problematic in contexts where early detection of fatigue is crucial. The *SVM* with a *Linear Kernel* presents a notable exception, with a markedly high recall rate, suggesting a potential utility in cases where the cost of missing true positives is particularly high, although at the expense of accruing more false positives. The lower performance of the *Gradient Boosting Classifier* could be indicative of model underfitting, which, along with the lowest accuracy observed in the *Light Gradient Boosting Machine*, suggests that these models may struggle with the complex patterns present in the multivariate time series data from IMUs. The *Logistic Regression*, *Linear Discriminant Analysis*, *Ridge Classifier*, and *Naive Bayes* models show a consistent pattern of high recall rates, which could be valuable in early fatigue detection but might also lead to a higher number of false alarms, a trade-off that must be carefully managed in a sports context. Interestingly, the *AdaBoost Classifier* demonstrates a close balance between precision and recall, yet its accuracy remains moderate. This balance might be beneficial in creating a baseline model upon which further fine-tuning could be conducted. Finally, the anomalously high performance of the *Dummy Classifier*, which classifies all instances as positive, serves as a cautionary note on the importance of evaluating model performance within the context of the specific application and the cost-benefit analysis of different types of prediction errors.

In summary, the results illustrate the nuanced performance of various classifiers in the context of AI-assisted sports performance (see Table 6, Table 7 and Table 8). They highlight the need for careful selection and tuning of models according to the specific demands and consequences of accurate or inaccurate predictions within the domain of athletic training and health management. The findings provide a foundation for further research, suggesting a potential for combining models or exploring more sophisticated algorithms to enhance prediction accuracy and reliability.

When dealing with inertial measurement unit (IMU)-generated multivariate time series datasets, Long Short-Term Memory (LSTM) networks often have advantages over traditional machine learning algorithms like Random Forest, Extra Trees Classifier, or Decision Tree Classifiers, and for this reason we conducted a dedicated LSTM deep learning (DL) experiment (see Table 9), taking into consideration that LSTM comes with challenges such as higher computational complexity, more intensive hyperparameter tuning, and a need for larger datasets to train effectively without overfitting, as well as that the success of LSTMs highly depends on the quality of data preprocessing, feature engineering. The LSTM model was trained with a significant number of epochs (5000), a batch size of 32, and utilized a validation split of 10% of the training data—a proportion of the data set aside for validation purposes is a standard practice, providing a means to monitor and evaluate the model’s performance on unseen data during training, thus helping in tuning and preventing overfitting. Our architecture included a DENSE output layer with two units and a softmax activation function, which is typical for binary classification tasks.

## 7. Results

Table 5 presents the comparison performance metrics of classifiers. Each row represents a different classifier algorithm, and the columns quantify their performance in terms of Accuracy, Precision, Recall, and F1 Score. Details:

1. Extra Trees Classifier ensemble learning method yields a moderate accuracy of 50.75%, with a slightly higher precision rate of 52.61%. Its recall rate is lower at 48.70%, which, combined with the precision, results in an F1 score of 50.22%. This indicates a balanced trade-off between precision and recall but suggests room for improvement in both specificity and sensitivity;
2. Random Forest Classifier ensemble method shows similar performance to the Extra Trees Classifier with an accuracy of 50.51% and a precision of 52.33%. The recall rate is slightly lower at 47.67%, leading to an F1 score of 49.57%, which points to a comparable performance with the Extra Trees Classifier but with a marginally lower recall;
3. Quadratic Discriminant Analysis classifier, based on a statistical approach, has a lower accuracy of 48.98% but a higher recall rate of 70.72%, indicating that it is better at identifying true positive cases. However, the precision is lower at 49.41%, leading to an F1 score of 52.76%, which is the highest among classifiers with an accuracy below 50%;
4. K-Nearest Neighbor Classifier, with an accuracy of 48.65% is an instance-based classifier and has a lower performance compared to tree-based methods. It also has a precision of 50.23% and a recall of 45.00%, resulting in an F1 score of 47.22%, which suggests a need for parameter tuning or consideration of alternative models;
5. Decision Tree Classifier shows a competitive accuracy of 50.66% and an F1 score of 51.15%. Its precision and recall are balanced at around 52%, indicating that it is a robust model, but not necessarily the most effective for this particular task;
6. Gradient Boosting Classifier has the lowest accuracy of 47.13% among the classifiers listed, with precision and recall rates near 49% and an F1 score of 45.90%. This indicates that the model might be underfitting or not capturing the complexity of the data well;
7. Logistic Regression and Linear Discriminant Analysis have similar accuracy rates of around 48.9%, with a higher recall rate exceeding 63% but lower precision around 48.5%. This high recall rate is reflected in a moderate F1 score of approximately 51%, suggesting that while these models are good at identifying positive cases, they may also be including a larger number of false positives;
8. AdaBoost Classifier shows a close balance between precision and recall, both approximately at 49%, reflected in an almost equal F1 score of 48.95%. The accuracy is slightly lower at 48.40%, indicating that it performs moderately across all metrics;
9. Ridge Classifier mirrors the performance of *Logistic Regression* and *LDA*, this classifier also demonstrates an accuracy and an F1 score of 48.81% and 51.08%, respectively. This suggests that these linear models may share a common strength and weakness in predicting the positive class but also misclassifying the negative cases;
10. Light Gradient Boosting Machine exhibits an accuracy of 49.58%, with a precision of 51.68% and a recall of 44.60%, leading to an F1 score of 47.16%. The model is somewhat precise but struggles with recall, suggesting that improvements are needed in identifying true positives;
11. SVM–Linear Kernel support vector machine model has an accuracy of 49.12%, with a notably high recall rate of 77.91% but a lower precision of 47.02%, which results in the highest F1 score of 54.39% among all models. This indicates a tendency of the model to favor recall over precision;
12. Naive Bayes is a probabilistic classifier and achieves an accuracy of 48.12% with a recall rate of 69.48% and a precision of 48.50%. Its F1 score of 52.09% is relatively high, considering the moderate accuracy, suggesting that it may be better at identifying true positives at the expense of accruing more false positives;
13. Dummy Classifier achieves an accuracy of 51.30%, with precision equal to accuracy, and a recall of 100%. This results in an F1 score of 67.78%, which is significantly higher than other classifiers. However, this high score is deceptive as it comes from a model that does not make real predictions but rather classifies all instances as the positive class, hence the perfect recall and disproportionate F1 score.

**Figure 7:**
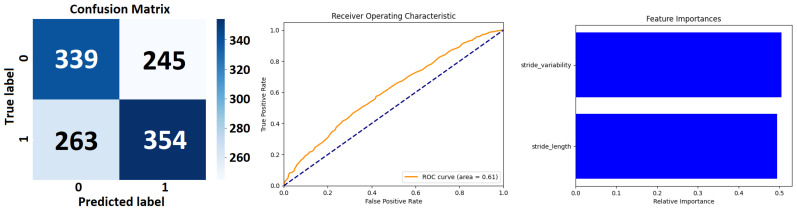
Extra Trees Classifier Experiment Results.

The *Extra Trees Classifier* (see Figure 7 and Table 6), configured with 100 estimators and a fixed random state for reproducibility has been applied to a binary classification task. The performance metrics of precision, recall, and F1 score are disaggregated for each class, alongside aggregate scores that encapsulate the model’s overall performance. The *precision* metric indicates the proportion of true positives against all positive calls made by the model.

For class 0, a precision of 0.56 suggests that 56% of the instances predicted as class 0 are indeed class 0. For class 1, the precision is slightly higher at 0.59, implying that this class is predicted with marginally better accuracy. *Recall*, or sensitivity, measures the model’s ability to identify all relevant instances. For class 0, the recall is 0.58, indicating that the model correctly identifies 58% of all actual class 0 instances. Class 1 has a recall of 0.57, meaning it correctly identifies 57% of all actual class 1 instances. The *F1 score* is the harmonic mean of precision and recall and serves as a single metric that balances both the false positives and false negatives. An F1 score of 0.57 for class 0 and 0.58 for class 1 suggests moderate agreement between precision and recall for both classes. The *model’s accuracy*, which is the ratio of correct predictions to total predictions, stands at 0.58. This indicates that the model correctly predicts the class of an instance 58% of the time across the dataset of 1201 instances. The weighted average precision, recall, and F1 score are all 0.58, indicating that when the model’s performance is weighed by the prevalence of each class in the data, the result is consistent with the macro average.

**Figure 8:**
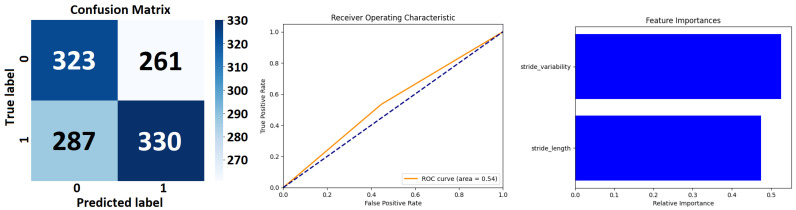
Decision Tree Classifier Results.

The class-specific performance of *Decision Tree Classifier* (see Figure 8 and Table 7) shows that for class 0, the precision is 0.53, which indicates that when the model predicts an instance to be class 0, it is correct 53% of the time. The recall for class 0 is slightly higher at 0.55, signifying that the model successfully identifies 55% of all actual class 0 instances within the dataset.

The F1 score, a balanced measure of precision and recall, is 0.54, suggesting a modest equilibrium between the two metrics for this class. For class 1, precision is marginally better at 0.56, indicating a slightly higher rate of correct predictions for the positive instances. However, the recall is 0.53, denoting that the model captures 53% of the actual class 1 instances. The F1 score for class 1 is 0.55, which is commensurate with the precision and recall values, again indicating a balanced performance, albeit slightly favoring precision over recall. The overall accuracy of the model is 0.54. This implies that, across both classes, the model correctly predicts the class of a given instance 54% of the time for the 1201 instances tested. The macro average for precision, recall, and F1 score is 0.54 across both classes. This average does not account for class imbalance and treats both classes with equal importance. A macro average of 0.54 indicates that, on average, the model’s performance is relatively uniform across both classes. The weighted average for precision, recall, and F1 score is also 0.54. Unlike the macro average, the weighted average takes into account the number of instances (support) for each class, thus adjusting the average for class imbalance. The fact that the weighted average, the macro average, and accuracy are all the same suggests that any class imbalance in the dataset does not have much of an effect on how well the model works.

**Figure 9:**
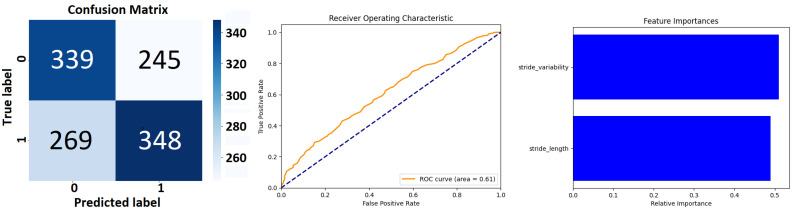
Random Forest Classifier Results.

Class-specific performance of a *Random Forest Classifier*’s performance (see Figure 9 and Table 8) shows that for class 0, the precision is 0.56. This indicates that 56% of instances predicted to be class 0 were correctly identified, suggesting a moderate ability of the classifier to discern true class 0 instances among all predicted as such.

The recall for class 0 stands at 0.58, meaning that the classifier correctly identified 58% of all actual class 0 instances, reflecting a slightly higher sensitivity compared to its precision. Class 1 exhibits a precision of 0.59, indicating that the model is slightly more precise in identifying true class 1 instances compared to class 0. The recall for class 1 is 0.56, which is slightly lower than that for class 0, suggesting that the classifier is slightly less sensitive in detecting all actual class 1 instances.

**Figure 10:**
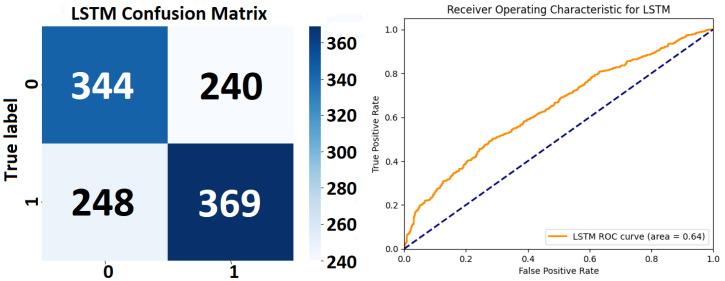
LSTM Results.

*Detailed Analysis of the LSTM Model’s Performance*: Class-Specific performance of the LSTM Model’s Performance Metrics (see Figure 10 and see Table 9) shows that for Class 0 the model attains a precision of 0.58, meaning that 58% of the predictions made as Class 0 are accurate. The recall, indicating the model’s ability to correctly identify actual instances of Class 0, is slightly higher at 0.59. This suggests a reasonably balanced detection of this class by the model. The F1 score, harmonizing precision and recall, stands at 0.58, reflecting a balanced compromise between the precision and recall rates. Class 1 exhibits a precision of 0.61, which is slightly higher than that for Class 0, indicating that the model is marginally more accurate in predicting Class 1 instances. The recall for Class 1 is 0.60, suggesting the model correctly identifies 60% of all actual instances of Class 1. The F1 score for this class is 0.60, mirroring precision and recall, indicating a similarly balanced performance but slightly favoring precision.

**Figure 11:**
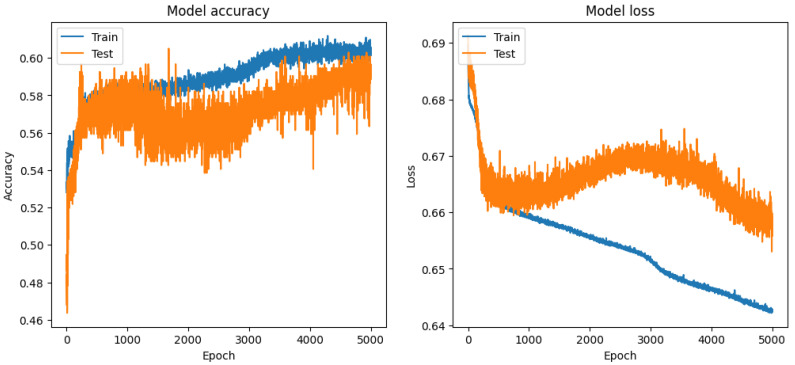
LSTM model accuracy (**left**) and model loss (**right**).

The overall accuracy of the model is 0.59, denoting that the model accurately predicts the class of an instance in 59% of cases across the dataset of 1201 instances. This level of accuracy is moderate, reflecting a fair degree of effectiveness in classification, yet also indicating room for improvement. The macro average, which treats both classes with equal importance, is 0.59 for both precision and recall. This uniformity in the macro average points to a consistent performance across both classes. The weighted average, taking into account the prevalence of each class, aligns with the macro average at 0.59 for precision, recall, and the F1 score. This congruence (see Figure 11) suggests a balanced performance across the classes, regardless of their representation in the dataset.

**Figure 12:**
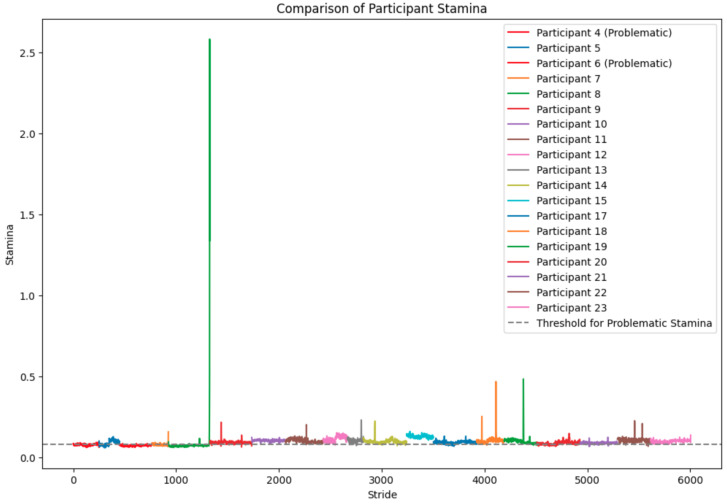
Comparison of participants’ stamina.

**Figure 13:**
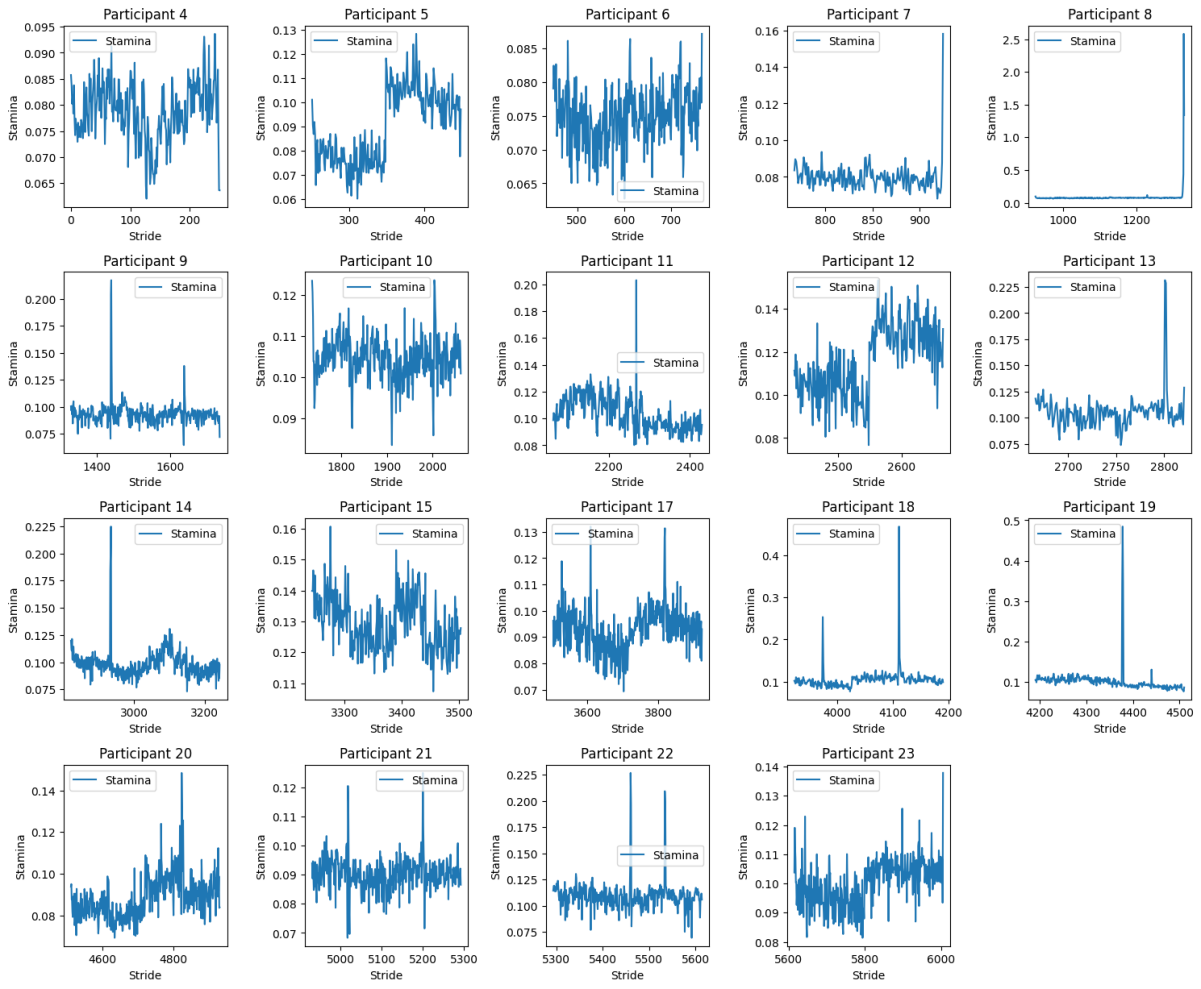
Stamina for analysis.

**Figure 14:**
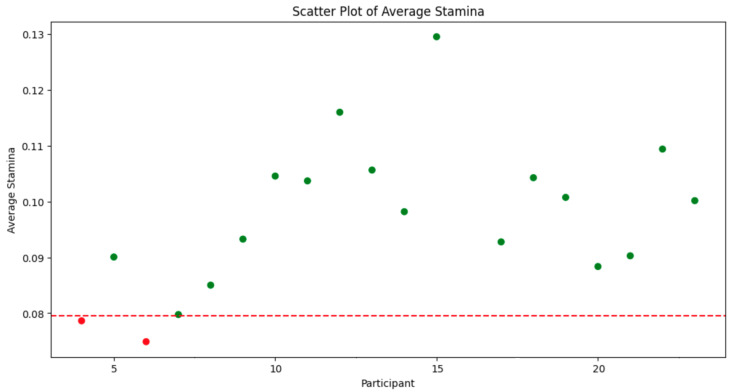
Scatter Plot of average stamina.

*Participant 4* exhibits relatively high fluctuations in stamina with a range of approximately 0.025 to 0.095, indicating considerable variability in their performance; *Participant 5* demonstrates moderate fluctuations in stamina values, yet overall less variability than Participant 4; *Participant 6* (see Figure 12) shows a dense clustering of stamina values with fewer peaks and troughs, suggesting a more consistent performance; *Participant 7* results indicate significant variability with some extremely high stamina values, which might suggest errors in data capture or exceptional moments of performance stability; (5) *Participant 8*: the stamina readings appear to be an anomaly, with values significantly higher than those of other participants (see Figure 13), possibly indicating a data recording error or an issue with the calculation of stamina for this individual; *Participant 9–Participant 23* display varying ranges of stamina, with some (such as *Participant 19*) having a particularly broad spread of values, suggesting inconsistent performance. Others, like *Participant 13*, have a tighter cluster of values, which may point to a more stable performance;

Overall Analysis: Variability in performance: the degree of fluctuation in stamina values across strides varies markedly between participants (see Figure 12). Some demonstrate tight clusters, indicative of consistent performance, while others have wide variances, suggesting fluctuations in their kinematic efficiency; Potential data anomalies: certain participants (e.g., Participant 8) show data patterns that deviate significantly from the norm, which may necessitate a review of data quality or methodological approaches specific to those cases; Identification of Outliers: participants such as 7 and 19, with wide-ranging stamina values, could be considered outliers in terms of the consistency of their running performance; Implications for training and rehabilitation: understanding stamina variability (see Figure 14) can inform tailored training programs to enhance consistency and efficiency in running. It may also aid in identifying the need for interventions to address specific kinematic deficits.

## 8. Discussion

The evaluation of classifiers presented in Table 5 suggests that no single model clearly outperforms the others across all metrics. The choice of the best classifier would depend on the specific requirements of the classification task at hand. For example, if the priority is to minimize false negatives, a model with a higher recall would be preferable. Conversely, if the focus is on minimizing false positives, a model with higher precision would be more suitable. The disparities in performance metrics underscore the need for a nuanced approach to classifier selection, possibly involving ensemble methods or further tuning of individual models.

The result from Table 6 of *Extra Trees Classifier* demonstrates a *balanced performance* between the two classes, with a slight variance favoring class 1 in precision and class 0 in recall. The model’s overall accuracy and average F1 score suggest that *it performs moderately well*, though there *may be room for improvement*, possibly through hyperparameter tuning or feature engineering. The consistency of the macro and weighted averages suggests that the model’s performance is stable across the classes, despite their slight differences in support within the dataset (see Figure 7).

The Decision Tree Classifier (see Table 7) demonstrates a balanced yet moderate performance in distinguishing between the two classes, with a slight propensity toward class 1 in terms of precision and toward class 0 in terms of recall. The overall accuracy, along with the macro and weighted averages, indicate a symmetrical performance across the classes. Given the decision tree’s inherent susceptibility to overfitting, the observed performance may also reflect this model’s limitation in generalizing from the training data to unseen data. Enhancements to the model, such as pruning, could potentially improve its predictive prowess. Additionally, the results may benefit from an examination of the decision tree’s depth and complexity, which could be contributing factors to its overall performance (see Figure 8).

The *RandomForestClassifier* as shown in Table 8 exhibits a balanced classification ability across both classes with moderate precision and recall. The uniformity of the macro and weighted averages indicates that the performance metrics are consistent across the classes. Given these results, the classifier appears to be a competent model for the dataset, though there is room for improvement. Potential steps to enhance the model’s performance could include hyperparameter tuning, feature selection, and engineering, as well as increasing the size or diversity of the training data. Additionally, depending on the application, different costs may be associated with false positives and false negatives, which could lead to adjustments in the model to favor either precision or recall (see Figure 9).

The research results reveal performance outcomes across various classifiers applied to AI-assisted fatigue and stamina control using IMU-generated multivariate time series datasets in performance sports. The *Extra Trees* and *Random Forest* classifiers, both tree-based ensemble methods, demonstrate moderate effectiveness, with accuracies slightly above 50% and balanced precision and recall rates. However, their comparable F1 scores of around 50% suggest a need for enhancement in both specificity and sensitivity, indicating that these models are proficient yet not optimal for this specific task. In contrast, the *Quadratic Discriminant Analysis* classifier, which relies on a statistical approach, shows an exciting trade-off: it achieves a lower accuracy, however, it excels in recall, suggesting that its strength lies in identifying true positive cases, albeit at the cost of precision. This is mirrored in its higher F1 score compared to other classifiers with sub-50% accuracy, indicating a potential for this model in scenarios where high recall is prioritized. The *K-Nearest Neighbor* and *Decision Tree* classifiers show lower performance metrics than the ensemble methods. Their lower F1 scores and balanced precision-recall suggest that these models might benefit from parameter optimization or may not be entirely suitable for the complex nature of the data. The *Gradient Boosting Classifier*’s lower performance metrics imply possible underfitting, indicating that this model might be overly simplistic for the intricate data patterns in the domain. *Logistic Regression*, *Linear Discriminant Analysis*, and *Ridge Classifier*, all linear models, exhibit similar patterns of higher recall rates but lower precision. This consistency suggests a common tendency in these models to favor identifying positive cases, even at the risk of increasing false positives. The *AdaBoost Classifier* presents a balanced performance but with overall moderate effectiveness, indicated by its nearly equal precision, recall, and F1 score, all hovering around 49%. The *Light Gradient Boosting Machine* demonstrates reasonable precision but struggles with recall, suggesting its efficacy in classifying true negatives but a need for improvement in detecting true positives. Notably, the *SVM* with a *Linear Kernel* stands out for its high recall rate and the highest F1 score among the models, implying a strong inclination toward identifying true positives, albeit at a compromise in precision. *Naive Bayes*, with its probabilistic approach, also tends to favor recall over precision, reflected in its relatively high F1 score despite moderate accuracy. Lastly, the *Dummy Classifier*, while exhibiting high accuracy and F1 score, serves as a reminder of the importance of interpreting these metrics in context. Its high scores are misleading as they result from a simplistic approach of classifying all instances as positive, underscoring the necessity of a nuanced understanding of model performance beyond superficial metrics.

These results highlight the complexities and trade-offs inherent in applying various AI classifiers to fatigue and stamina prediction in sports. The findings underscore not only the importance of dataset preparation, but also the need for careful model selection and optimization tailored to the specific requirements and nuances of the sports health domain.

The LSTM model (see Table 9), with its extensive training regime, demonstrates a consistent and balanced performance (see Figure 10) in classifying both classes, with a slight edge in precision for Class 1. The similar precision and recall metrics across the classes suggest an unbiased model performance. However, the overall accuracy of 0.59, while reasonable, suggests potential areas for enhancement. Improvements might include further hyperparameter tuning (adjusting the number of LSTM units, changing the learning rate, or experimenting with different activation functions), revisiting the feature engineering process, or employing techniques to combat overfitting, which might be a concern given the high number of training epochs. The high number of epochs in the training process could also raise concerns about the computational efficiency and the risk of overfitting, particularly if validation accuracy plateaued early in the training process. Therefore, monitoring the model’s performance on the validation set throughout the training process is crucial in optimizing epoch count and preventing overtraining (see Figure 11).

Overall lower medians and more variability in the “Fatigued” state compared to the “Not Fatigued” state are indications that fatigue negatively affects the acceleration characteristics of participants, according to the acceleration distribution box plots. These findings could have implications for designing training regimens, where monitoring acceleration could serve as a proxy for fatigue levels and, hence, the effectiveness of the training session. Further statistical analysis would be required to confirm these observations and understand the underlying factors contributing to the observed patterns. On the other hand, stamina plots suggest that there is a significant inter-individual variation in the kinematic consistency of runners as measured by stamina across strides. The findings underscore the need for personalized approaches to performance monitoring and coaching. More research into the biomechanical and physiological causes of these differences could give us more information about the endurance profiles of regular runners. Additionally, verifying the accuracy of the anomalous data points is crucial for ensuring the integrity of the study’s conclusions.

### 8.1. Importance of the Research and Applicability to Diverse Disciplines

The research results on AI-supported management of fatigue and stamina in competitive sports, using IMU-generated time series datasets, have substantial relevance outside of the sports domain, particularly in the areas of physical therapy and rehabilitation. The accuracy of AI in identifying movement patterns can play a crucial role in evaluating patients’ progress during physical therapy. This enables therapists to customize rehabilitation activities according to each individual’s specific recovery requirements. AI’s predictive abilities, developed in sports to detect weariness early, modified patterns, or anti-patterns, can be applied to prevent excessive strain in patients during rehabilitation, reducing the likelihood of injury relapse or worsening.

Additionally, AI-supported IMU technology’s ongoing surveillance enables therapists to receive immediate feedback on patients’ biomechanical responses to therapy, enhancing the efficacy of treatment plans. AI models can be utilized in post-operative rehabilitation to forecast recovery trajectories, facilitating more precise prognoses and tailored rehabilitation regimens. The data-driven technique can also detect small enhancements or declines in patients’ conditions that may be disregarded in traditional evaluations, resulting in prompt actions. Moreover, the implementation of this research in rehabilitative settings highlights the capacity of AI and wearable technology to manage chronic pain and improve mobility. This strategy relies on data analysis to provide patient care. The use of these AI approaches across several disciplines holds the potential to completely transform rehabilitation practices, making a substantial contribution to the recovery of patients and enhancing their overall quality of life. The novelties also highlight the importance of multimodal learning, which will connect different domains and fields.

### 8.2. Ethical Considerations

Using AI technology to tailor training programs (revealing hidden patterns and segments that can be improved or fine-tuned) and enhance performance results for athletes raises many ethical concerns. First and foremost, the issue of data privacy and security arises due to the need for AI systems to gather and analyze athletes’ sensitive personal and physiological data. The possibility of improper use or unlawful entry of these data prompts concerns regarding safeguarding athlete confidentiality and the administration of data ownership. Furthermore, there is a potential for an inequitable competitive edge to arise and maybe this needs more careful focus from legislators within performance sports, as the availability of sophisticated AI technologies may be restricted to athletes or teams with more significant financial means, exacerbating the disparity between affluent and underfunded sports programs. In addition, the dependence on AI for improving performance may unintentionally devalue the significance of natural talent and diligent effort, potentially resulting in an excessive focus on technological intervention in sports. Adding AI to sports also raises concerns about the independence of athletes and teams, since decisions influenced by AI analytics may take precedence over athletes’ personal preferences or gut feelings. This could change the traditional relationship between a coach and an athlete. The ethical consequences of using AI in sports require thorough examination and regulatory supervision to guarantee that its implementation adheres to the norms of equity, confidentiality, and the preservation of the athlete’s independence.

### 8.3. Limitations of the Approach

Although AI-assisted fatigue and stamina control in performance sports has made some progress, there are still some problems and limitations that need to be carefully thought through when using IMU-generated multivariate time series datasets. Firstly, the quality and granularity of data collected by IMUs are crucial. IMUs can be susceptible to noise and errors in data capture, which can lead to inaccuracies in the AI’s analysis (see Table 2) and subsequent recommendations. The accuracy of the sensors, where they are placed on the athlete’s body, and the algorithms used to clean and process the data [58] can all make the method less useful. Moreover, the interpretation of AI-generated insights requires domain expertise. Coaches and sports scientists must understand the context and limitations of the data. Over-reliance on AI without a nuanced understanding of an athlete’s physical and psychological state could lead to suboptimal or even detrimental training decisions. The individual variability among athletes presents another challenge. AI models often require large amounts of data to accurately predict fatigue and stamina thresholds. However, the unique physiological and biomechanical profiles of each athlete mean that models must be highly personalized, which can be data-intensive and time-consuming to develop. There is also the risk of overfitting, where an AI model may perform exceptionally well on historical data but fail to generalize to new or unseen data. This can occur when the model learns the noise within the dataset rather than the underlying pattern it is meant to predict.

Additionally, due to the dynamic nature of human physiology, psychological stress, sleep quality, nutrition, and other lifestyle factors can all have an impact on an athlete’s level of fatigue and stamina. The exclusion of these factors can limit the comprehensiveness of AI assessments. Ethical considerations also come into play. The pervasive monitoring of athletes raises privacy concerns, and the data must be handled with strict confidentiality and consent protocols. Lastly, there is the inherent resistance to change within established training paradigms. Coaches and sports professionals may be hesitant to adopt AI-driven methods, preferring traditional training and recovery methodologies. This resistance can slow down the integration of AI technologies into athletic training and management.

Overall, AI-assisted fatigue and stamina control using IMU-generated data has a lot of potential to improve athletic performance. However, it is limited by technological, methodological, and ethical issues that need to be solved by technologists, sports scientists, coaches, and athletes working together.

## 9. Future Development

The study of AI-assisted fatigue and stamina control for performance sports, which is based on analyzing IMU-generated multivariate time series datasets, is set to lead to new discoveries. The prospective development lines and innovative outcomes of such research are certain to significantly influence sports science, athlete training, and broader AI applications. Potential future development lines are the following:

1. *Algorithmic advancement and hardening*: Future research is expected to advance the sophistication of algorithms, potentially incorporating hybrid models that blend ML with biomechanical simulations to yield even more accurate predictions of fatigue and stamina. An integrated platform that combines fatigue and stamina management with nutrition, sleep, and mental health could emerge, providing athletes with a comprehensive resource for all aspects of training and recovery.
2. *Sensor integration*: The integration of additional sensor technologies, such as heart rate variability monitors and biochemical sensors, could enhance the multidimensional aspect of the datasets, providing a more holistic view of an athlete’s physiological state. The seamless integration of IMUs and AI analytics into wearable technology could lead to the development of smart apparel that not only tracks performance but also offers real-time feedback and training adjustments.

In summary, the prospective research on AI-assisted fatigue and stamina control, harnessing the power of IMU-generated multivariate time series datasets, is on the cusp of fostering transformative outcomes for athletic training. The convergence of AI, sensor technology, and personalized medicine holds the promise of revolutionizing performance sports, with implications that extend into the health and wellness industries, personalized healthcare, and beyond.

## 10. Conclusions

Implementing a precognition model for AI-assisted fatigue and stamina control (see Figure 1) in performance sports, derived from inertial measurement unit (IMU)-generated multivariate time series data, yields several substantial conclusions with far-reaching implications on sports science and athlete performance management. In conclusion, the LSTM model (see Table 9) exhibits a competent capability in handling the classification task at hand, with its performance suggesting a balanced approach to classifying the binary classes. However, given the complexity and the “black box” nature of LSTM models, a careful examination and potential comparison with other machine learning models, including simpler ones, would be prudent, particularly in contexts where interpretability and computational efficiency are crucial.

Firstly, the precision of fatigue detection is a salient conclusion. The model’s ability to discern nuanced physiological changes, which precede observable signs of fatigue (see Figure 6), represents a form of early detection system. It facilitates interventions at the incipient stages of fatigue, thereby preempting the negative impacts on muscle performance and coordination that can result in injuries or suboptimal training outcomes. Secondly, the model reinforces the importance of individualized athlete profiles. By recognizing patterns over time (see Figure 5), AI can distinguish between an athlete’s normal variability in performance versus genuine markers of fatigue or improvements in stamina. This longitudinal tracking allows for the tailoring of training loads and recovery times, which are responsive to the athlete’s personal physiological cycles, such as circadian rhythms and adaptive responses to training stimuli. Furthermore, the model elucidates the correlation between various biomechanical metrics and their collective impact on athlete performance. For example, the analysis of stride length (see Figure 13), cadence, and ground contact time in runners may reveal specific fatigue signatures that inform about the athlete’s current stamina level (see Figure 12) and biomechanical efficiency. This insight enables the optimization of both technique and physical conditioning to enhance performance endurance.

Additionally, the application of the precognition model extends to optimizing rest periods and recovery protocols. By predicting the trajectory of an athlete’s recovery process, the model provides evidence-based recommendations for rest durations and recovery activities that are custom-fitted to the athlete’s physiological needs, thus maximizing recovery efficiency and effectiveness. Another significant conclusion is the model’s contribution to strategic planning in training and competition. Predictive insights into an athlete’s stamina (see Figure 14) and fatigue thresholds enable coaches to make informed decisions regarding training intensity, competition schedules, and even strategic in-competition pacing strategies.

The model also highlights the potential of AI in managing the balance between training and recovery. By continuously adjusting to the athlete’s current state, the model operates as a dynamic regulatory system, akin to a physiological thermostat, maintaining training within the optimal zone for performance gains while avoiding overtraining. Finally, the implementation of this precognition model demonstrates the transformative potential of ML and AI in the domain of high-performance sports. It not only enhances the scientific rigor of training practices but also ushers in a new era of data-driven, precision sports medicine. The model’s predictive capabilities, grounded in high-resolution multivariate time series data, exemplify a pioneering step toward the future of athletic training, where AI’s role is central to fostering peak performance and athlete well-being.

Incorporating inertial measurement units (IMUs) for real-time, AI-supported monitoring and predicting specific information in current sports training and competition regimens represents notable progress in precision athletics. These sensors connect to athletes’ equipment and give essential biomechanical information such as acceleration and angular velocity. By using AI on top of these data, it allows non-invasive tracking and fine-tuning of performance. Coaches who have received training in data interpretation can utilize this information to provide prompt feedback, optimize the intensity and technique of the training in real time, and improve the effectiveness of the sessions while avoiding excessive strain. IMU data are valuable in competitive situations as it helps inform strategic decision-making and post-event analysis. It provides useful information that can be used to improve performance and make tactical adjustments. The technology’s uninterrupted flow of data enables the construction of customized training plans specifically designed to match the distinct physiological characteristics of each athlete, thereby promoting more efficient and personalized progress. Implementing robust data management procedures is crucial, especially for upholding privacy and enforcing consistent data processing methods.

Effective utilization of IMU with AI requires essential collaboration among data scientists, sports medicine practitioners, and coaching personnel. In summary, incorporating IMUs into sports protocols enhances athletic performance. It contributes substantially to athlete well-being and the length of their careers, representing the trend toward sports science based on data analysis.

## Figures and Tables

**Table 1:** Contrasting the challenges in sports health with the solutions provided by classic methods and those provided by AI-assisted approaches.

Challenges/Issues	Existing Solutions [7,8]	Novelties of the AI-Assisted Approach
Fatigue detection	Reliant on subjective measures, such as athlete self-reports and coach observations.	AI models predict fatigue objectively before physical symptoms manifest, using physiological data.
Personalization	Generic training programs, one-size-fits-all approach with limited personalization.	Tailored training regimens adapted to individual physiological responses and recovery profiles.
Real-time feedback	BDelayed feedback after training sessions, based on manual data review.	Instantaneous feedback during training via wearable tech integration, enabling immediate adjustments.
Injury prevention	Reactive approaches that respond to injuries post-occurrence.	Proactive injury risk assessments and preventative suggestions based on predictive analytics.
Training load optimization	Empirical methods for deciding on training loads, often leading to over- or under-training.	Data-driven load optimization that continuously adapts to an athlete’s current state and needs.
Long-term monitoring	Fragmented data collection with sporadic athlete testing, lacking continuity.	Continuous monitoring and longitudinal performance tracking, with detailed historical data analysis.

**Table 2:** Comparison between existing solutions and AI-assisted approaches for fatigue and stamina control in performance sports.

Aspect	Existing Solutions [7,8]	AI-Assisted Approach
Simplicity and accessibility	Rely on easily interpretable metrics like heart rate; less technical expertise required.	May require specialized knowledge to interpret Complex AI model outputs.
Proven frameworks	Backed by empirical research and traditional sports science principles.	Emerging, potentially backed by data science and machine learning (ML) principles.
Ease of implementation	Can be implemented without complex infrastructure.	Requires significant investment in data collection and processing infrastructure.
Data utilization	Limited to simple and fewer data points; may miss multifaceted fatigue indicators.	Utilizes large, multivariate datasets from IMUs for a comprehensive analysis.
Predictive capabilities	Generally reactive, identifying issues after after they occur.	Predictive, with the potential to preemptively adjust training to prevent fatigue.
Personalization	Often generic, leading to one-size-fits-all training programs.	Highly personalized, with tailored training and recovery recommendations.
Continuous learning	Static models with limited adaptability to new data.	Models improve over time with continuous learning and data input.
Infrastructure requirements	Minimal technical requirements for data collection and analysis.	Requires advanced systems for real-time data collection and ML analysis.

**Table 3:** Comparison between physiological and ai-assisted methods for athlete fatigue and stamina assessment: physiological versus kinematic data comparison.

Aspect	Physiological Methods	AI-Assisted Methods
Data collection	Invasive and episodic	Non-invasive and continuous
Real-time analysis	Limited	Enabled
Cost	Often high	Potentially lower
Accessibility	Specialized facilities	Broad accessibility
Precision	High for single assessments	Developing for continuous monitoring
Frequency	Periodic testing	Continuous feedback
Invasiveness	Typically high	Low
user experience	Can be disruptive	Minimally disruptive
Validation	Established	Ongoing

**Table 4:** Comparison between video-based analysis and AI-assisted methods with IMUs for athlete fatigue and stamina assessment.

Aspect	Video-Based Analysis (Gold Standard)	AI-Assisted Methods with IMUs
Data collection	Manual recording	Automated and continuous
Real-time analysis	Typically delayed	Enabled and instantaneous
Cost	Resource-intensive for analysis	Cost-effective in the long-term
Accessibility	Requires presence and setup	Portable and accessible in various settings
Precision	High with expert analysis	High with advanced algorithms
Frequency	During specific sessions	Throughout training and competition
Invasiveness	Non-invasive but can be obtrusive	Non-invasive and unobtrusive
User experience	Potentially interruptive	Seamless integration into gear
Data richness	Limited to visual capture	Multidimensional kinematic data
Analytical depth	Subject to analyst’s expertise	Deep, algorithm-driven insights
Validation	Well-established	Subject to ongoing research

**Table 5:** Results with most well-known classifiers.

No.	Classifier	Accuracy	Precision	Recall	F1 Score
0	Extra Trees Classifier	0.507515	0.526137	0.486982	0.502192
1	Random Forest Classifier	0.505092	0.523278	0.476686	0.495675
2	Quadratic Discriminant Analysis	0.489837	0.494120	0.707237	0.527557
3	K-Nearest Neighbor Classifier	0.486452	0.502285	0.450020	0.472184
4	Decision Tree Classifier	0.506605	0.521075	0.505028	0.511507
5	Gradient Boosting Classifier	0.471260	0.495196	0.451750	0.459049
6	Logistic Regression	0.489593	0.485458	0.630432	0.510876
7	AdaBoost Classifier	0.484047	0.498580	0.489276	0.489536
8	Linear Discriminant Analysis	0.488134	0.483434	0.633499	0.510848
9	Ridge Classifier	0.488134	0.483434	0.633499	0.510848
10	Light Gradient Boosting Machine	0.495775	0.516807	0.445978	0.471630
11	SVM—Linear Kernel	0.491180	0.470230	0.779147	0.543881
12	Naive Bayes	0.481170	0.485005	0.694849	0.520882
13	Dummy Classifier	0.512985	0.512985	1.000000	0.677771

**Table 6:** Results with ExtraTreesClassifier (parameters: n_estimators = 100, random_state = 42).

Class	Precision	Recall	F1 Score	Support
0	0.56	0.58	0.57	584
1	0.59	0.57	0.58	617
accuracy			0.58	1201
macro avg	0.58	0.58	0.58	1201
weighted avg	0.58	0.58	0.58	1201

**Table 7:** Results with DecisionTreeClassifier (parameters: random_state = 42).

Class	Precision	Recall	F1 Score	Support
0	0.53	0.55	0.54	584
1	0.56	0.53	0.55	617
accuracy			0.54	1201
macro avg	0.54	0.54	0.54	1201
weighted avg	0.54	0.54	0.54	1201

**Table 8:** Results with RandomForestClassifier (parameters: n_estimators = 100, random_state = 42).

Class	Precision	Recall	F1 Score	Support
0	0.56	0.57	0.56	584
1	0.58	0.57	0.58	617
accuracy			0.57	1201
macro avg	0.57	0.57	0.57	1201
weighted avg	0.57	0.57	0.57	1201

**Table 9:** Results with the LSTM (Long Short-Term Memory) deep learning network (parameters: epochs = 5000, batch_size = 32, validation_split = 0.1), with the DENSE layer (parameters: units = 2, activation = ’softmax’).

Class	Precision	Recall	F1 Score	Support
0	0.58	0.59	0.59	584
1	0.61	0.60	0.60	617
accuracy			0.59	1201
macro avg	0.59	0.59	0.59	1201
weighted avg	0.59	0.59	0.59	1201

## Data Availability

The dataset used in this experiment is known as the “Multivariate Time Series data of Fatigued and Non-Fatigued Running from Inertial Measurement Units (0.0) [Data set]” and is freely available for academic research; there are no (legal or other) constraints on using the data for scientific purposes [39]. The data are available upon request. The figures, architecture, dataset, and results charts of this study can be found at https://doi.org/10.6084/m9.figshare.24615450 (accessed on 25 November 2023).

## Abbreviations

The following abbreviations are used in this manuscript:

IMU	inertial measurement unit
AI	artificial intelligence
LSTM	Long Short-Term Memory Networks
ML	machine learning
DNN	deep neural network
GRU	gated recurrent unit
NN	neural network
DL	deep learning
BLE	Bluetooth Low Energy
MPC	Model Predictive Control
RL	Reinforcement Learning
IQR	interquartile range
QDA	Quadratic Discriminant Analysis
KNN	K-Nearest Neighbor
PSD	power spectral density
FFT	fast Fourier transform
DTW	dynamic time warping
CNN	convolutional neural network
SMA	signal magnitude area
